# Differential effects of tau expression on seizures and epileptogenesis in a mouse model of temporal lobe epilepsy

**DOI:** 10.3389/fnsys.2025.1693339

**Published:** 2025-12-18

**Authors:** Madeleine C. Moseley, Ryan A. Cloyd, Liam Burns, Rafael Roberts, Young-Jin Kang, Sang-Hun Lee, Bret N. Smith

**Affiliations:** 1 Department of Biomedical Sciences, Colorado State University, Fort Collins, CO, United States; 2 Department of Physiology, University of Kentucky College of Medicine, Lexington, KY, United States; 3 Department of Neuroscience, University of Kentucky College of Medicine, Lexington, KY, United States

**Keywords:** electroencephalography (EEG), excitatory postsynaptic current (EPSC), inhibitory postsynaptic current (IPSC), intrahippocampal kainate, tau, temporal lobe epilepsy (TLE)

## Abstract

Studies of the microtubule-associated protein, tau suggest its promise as a potential target for epilepsy disease modification, but mechanisms underlying tau’s effects on seizures are not well-defined. Acquired temporal lobe epilepsy (TLE) is the most prevalent form of focal epilepsy, yet the impact of tau expression on the process of TLE development is unexplored. We investigated tau’s role in the epileptogenesis of acquired TLE using the intrahippocampal kainate (IHK) model in mice lacking tau expression (i.e., tau^−/−^ mice). We examined epileptiform activity during status epilepticus (SE) after IHK injection and assessed the subsequent development of spontaneous recurrent seizures (SRS) using video and video-electroencephalography (v-EEG). Results demonstrate that the lack of tau expression did not prevent evoked seizures or the development of TLE but reduced the number of convulsive seizures during SE and the severity of spontaneous seizures after TLE developed by suppressing epileptiform electrographic activity of convulsive seizures, which has not been shown in the context of an acquired TLE model. We assayed excitatory and inhibitory synaptic properties of dentate granule cells (DGCs) in the dorsal hippocampus using whole-cell patch clamp electrophysiology once TLE developed. Our results show that DGCs in tau^−/−^ mice receive significantly fewer spontaneous inhibitory synaptic current events than in wildtype controls and, after tau^−/−^ mice develop TLE, DGCs develop increased contralateral inhibitory input. The modified inhibitory synaptic neuroplasticity associated with acquired TLE development, which is consistent with altered EEG spectra during convulsive seizures, may contribute to modified spontaneous seizure expression. Deletion of tau expression therefore modifies seizure expression, potentially via mechanisms involving inhibitory synaptic circuits in the dentate gyrus but does not prevent epileptogenesis in a murine model of acquired TLE.

## Introduction

Temporal lobe epilepsy (TLE) is the most common focal epilepsy and accounts for a majority of drug-resistant epilepsy cases (Semah et al., 1998; Reynolds, 2000; Wiebe, 2000; Pascual, 2007; Asadi-Pooya et al., 2017). Although drug resistant TLE with an identifiable focus often responds well to surgical resection (Engel, 1996; Engel et al., 2003; Engel et al., 2012), development of additional treatment options remains essential to reduce the burden of care and improve quality of life for patients with TLE. Further, there are no approved antiepileptogenic therapies for TLE and improved understanding of the mechanisms that drive TLE development will be crucial to developing therapies to prevent epileptogenesis. One potential mechanistic target that has received attention in recent years is the microtubule-associated protein, tau.

Tau protein is highly expressed in the hippocampus where it plays important roles in microtubule stabilization and development of hippocampal neurons (Dawson et al., 2001; McMillan et al., 2008; Cantero et al., 2011). Mice lacking tau expression function normally with no physiological deficits, but exhibit modified neuronal maturation and altered synaptic activity leading to suppression of network hypersynchrony (Dawson et al., 2001; Tucker et al., 2001; Roberson et al., 2007; Chang et al., 2021). Additionally, we previously identified age dependent, transient effects of tau deletion on excitability of dentate granule cells (DGCs) (Cloyd et al., 2021).

Genetic deletion or suppression of tau expression with antisense oligonucleotides improves seizure outcomes in animal models of genetic epilepsies (i.e., channelopathies) and chemoconvulsant models of evoked seizures (Roberson et al., 2007; DeVos et al., 2013; Holth et al., 2013; Gheyara et al., 2014; Li et al., 2014; Putra et al., 2020; Shao et al., 2022). However, tau’s role in acquired TLE remains unclear. This is important because the etiologies of genetic and acquired epilepsies are vastly different. Spontaneous seizures in acquired epilepsies often develop after a delay following an injury (e.g., status epilepticus (SE), stroke, traumatic brain injury) after which cortical circuits become rewired through synaptic reorganization, resulting in an increased probability of unprovoked seizures. Unlike many genetic epilepsies, synaptic reorganization associated with TLE development is characterized by axon sprouting of dentate granule cells (DGCs) and other neurons and selective neuron loss, including subsets of inhibitory interneurons, which ultimately decreases synaptic inhibition and increases recurrent excitation within the dentate gyrus (Cronin et al., 1992; Buckmaster et al., 2002). Although resistance to evoked seizures has been demonstrated in animals lacking tau (Putra et al., 2020), whether this translates to altered epileptogenesis is not clear and the mechanisms by which tau impacts seizures and epilepsy are not known (Chang et al., 2021). Furthermore, the degree to which tau promotes seizures and epileptogenesis in the absence of additional, underlying pathology (e.g., channelopathy) has not been adequately studied. Studies have examined the link between tau hyperphosphorylation and epileptogenesis using animal models of TLE, where insults also lead to tau hyperphosphorylation, at least transiently (Canet et al., 2021; Concepcion et al., 2023) and dephosphorylating tau may modify evoked seizures and disease progression in animal models of TLE (Jones et al., 2012; Liu et al., 2016; Casillas-Espinosa et al., 2023). Even so, few studies have examined how tau deletion alters excitability and network activity of DGCs (Cloyd et al., 2021) and none have examined how this relates to epileptogenesis in a mouse model of acquired TLE.

Here, we used the intrahippocampal kainate (IHK) model of acquired TLE to assess epileptiform activity and convulsive seizure expression during SE and the subsequent development of spontaneous recurrent seizures (SRS) using video and video-electroencephalography (v-EEG) in mice lacking tau expression (i.e., tau^−/−^ mice) and in those expressing native murine tau (i.e., wildtype). We further examined synaptic transmission in DGCs because of their involvement in epileptogenesis and tauopathy-associated pathology (Hunt et al., 2010; Butler et al., 2015; Cloyd et al., 2021). We tested the hypothesis that tau deletion confers evoked seizure resistance and suppresses—but does not prevent—the process of epileptogenesis in acquired TLE.

## Materials and methods

### Animals

Transgenic B6. Cg-Mapttm1(EGFP)KltTg(MAPT)8cPdav/J mice were produced in house from breeders obtained from The Jackson Laboratory (JAX; Bar Harbor, ME; stock #005491). This mouse strain was originally generated on a hybrid Swiss Webster/B6D2F1 hybrid background but has been backcrossed to C57BL/6 J for more than 10 generations. Single nucleotide polymorphism (SNP) analyses performed by JAX were consistent with a pure C57BL/6 J background, which served as the control strain. All breeding mice were homozygous for the deletion of the murine tau gene. One mouse in each breeding pair was hemizygous for a transgene expressing all six isoforms of non-mutant, human tau protein. The offspring are therefore either full tau knockout (tau^−/−^) or express only human tau (htau), and genotypes and protein expression phenotypes were confirmed in our recent report (Cloyd et al., 2021). Here, we used tau^−/−^ mice and isogenic C57BL/6 J wildtype control mice that express murine tau protein.

DNA was extracted from tail snips and genotype was confirmed via PCR according to the protocols supplied by JAX. Disruption of the endogenous murine tau gene was confirmed using the primer pair 5′-CGTTGTGGCTGTTGTAGTTG-3′ and 5′-TCGTGACCACCCTGACCTAC-3′, which amplifies a fragment at 270 bp in tau^−/−^ mice. Age matched male C57BL/6 J control mice were bred in house from breeders obtained from JAX (#000664). All mice were housed under a 12 h light/12 h dark cycle in an Association for Assessment and Accreditation of Laboratory Animal Care (AALAC) approved facility. Food and water were available ad libitum. Colorado State University and University of Kentucky Laboratory Animal and Use Committees approved all procedures. Principles outlined in the Animal Research: Reporting of *in Vivo* Experiments (ARRIVE) guidelines and the Basel declaration, including the 3R (Replacement, Reduction, Refinement) concept, were considered when planning the experiments.

### IHK mouse model of TLE

All surgical procedures were performed under isoflurane general anesthesia with 0.05% bupivacaine local anesthesia. Kainic acid (120 nL, 20 mM in 0.9% saline, Tocris Bioscience; Minneapolis, MN) or saline (sham; 120 nL, 0.9% saline) was injected using a syringe pump (Harvard Apparatus Pump 11 Elite; Holliston, MA) into the left dorsal hippocampus (2.0 mm posterior, 1.25 mm left, and 1.6 mm ventral to bregma) in male and female tau^−/−^ and wildtype mice between 6 and 8 weeks of age (Krook-Magnuson et al., 2013). The injection rate was 100 nL/min, and the needle (Hamilton syringe) was left in place for 5 min before and after injection. Buprenorphine SR (0.05 mg/kg) was administered subcutaneously after surgery. Mice were transferred to a heated cage for recovery and monitored for convulsive behavioral seizures for 4 h to assess development of SE. SE was defined as the occurrence of at least 3 convulsive seizures of Racine scale 3 or higher during the observation period, since convulsive seizures during SE predict the successful conversion to TLE within 2 months in C57BL/5 J mice (Racine, 1972; Shibley and Smith, 2002; Kang et al., 2021). After 4 h post-IHK or vehicle, diazepam (7.5 mg/kg) was administered intraperitoneally to terminate SE.

### Video and video-EEG monitoring during SE induction and development of spontaneous seizures

Randomly selected cohorts of mice were fitted with surface EEG head mounts to allow for v-EEG recording of seizures during the SE induction period and for up to 3 weeks after IHK or vehicle injection. Kainic acid or saline was injected as described above. One channel screw with wire lead was placed 2.0 mm left of midline and 1.0 mm posterior to bregma. Ground and reference screws with wire leads were placed 2.0 mm left and right of midline and 5.0 mm posterior to bregma. Once screws were in place, a tethered 6-Pin Surface Mount Mouse Head mount EEG (Pinnacle Technology model #8235-SM; 2,721 Oregon St., Lawrence, KS 66046) was soldered to wire leads and secured with dental cement. Mice were left to recover from anesthesia as described above. v-EEG recording began ~1 h after surgery and continued until the administration of diazepam as described above and again for 72 h beginning 3 wks after SE induction. Recordings were sampled at 2 kHz and high pass filtered at 1 kHz and low pass filtered at 0.1 kHz.

The same cohorts fitted with surface EEG head mounts and additional cohorts without EEG head mounts were monitored for spontaneous seizures by video-EEG or video 3 weeks after the IHK or vehicle injection. Each animal underwent a minimum of one recording session of 72 continuous hours. The videos were reviewed at 4x speed by an investigator(s) blind to genotype and treatment; all seizures were verified by a second investigator. Mice that exhibited at least one spontaneous behavioral and electrographic seizure during the recording period were defined as developing TLE.

Electrographic seizures were analyzed with Neuroscore (Data Sciences International; DSI; St. Paul, MN). Electrographic seizures were confirmed by behavioral assessment of convulsive seizures (≥ 30 s duration) using a modified Racine rating system analysis for mice (i.e., Racine 3–5; Racine 3: unilateral forelimb myoclonus; Racine 4: bilateral forelimb myoclonus; Racine 5: postural instability and/or repetitive jumping). Quantification of epileptiform spikes (i.e., spike frequency) during convulsive seizures were defined by a threshold ratio of 2 from the baseline signal. Seizure onset was defined as behavioral signs (i.e., Racine scale) with concurrent electrographic epileptiform activity exceeding twice the baseline signal amplitude. Termination was marked by cessation of behavioral seizure hallmarks and the onset of postictal electrographic activity (≤ 0.5 μV of baseline signal). Duration of convulsive seizures, either during SE or spontaneously, was defined by seizure onset and termination as described above. Mice that did not exhibit convulsive seizures during SE induction demonstrated high amplitude, low frequency electrographic spiking therefore spike frequencies (i.e., spikes/s) in mice that did not exhibit convulsive SE was calculated over 1 h at similar times post IHK injection. For spectral analysis, electrographic seizures were defined like above, segmented into 10 s epochs and subjected to fast Fourier transformation (FFT) for the estimation of power spectral density from 0.5–100 Hz with standard frequency bands being defined as: delta: 0.5–4 Hz, theta: 4–8 Hz, alpha: 8–12 Hz, sigma: 12–16 Hz, beta: 16–30 Hz, and gamma: 30–100 Hz. Periodogram analysis allowed for power estimates at each frequency. Slower background spikes (e.g., movement artifacts) were disregarded during analysis.

### Whole-cell patch clamp electrophysiological recordings

Coronal dorsal hippocampal slices (300 μM) were prepared from vehicle and IHK-treated tau^−/−^ and wildtype mice 6–8 weeks after IHK or saline injection. After cutting in cold, oxygenated sucrose artificial cerebrospinal fluid (ACSF), slices were incubated for 1 h at 34 °C then held at room temperature until recordings. Slices were transferred to a recording chamber containing ACSF (in mM): 124 NaCl, 26 NaHCO_3_, 11 glucose, 3 KCl, 2 CaCl_2_, 1.3 MgCl_2_, and 1.4 NaH_2_PO_4_; 32-34 °C. Slices were visualized with an upright microscope (BX51W1, Olympus) with infrared-differential interference contract optics.

Whole-cell patch-clamp recordings were obtained from hippocampal DGCs identified by location and morphological characteristics. To examine synaptic properties of DGCs, spontaneous excitatory post-synaptic currents (sEPSCs) and spontaneous inhibitory post-synaptic currents (sIPSCs) were recorded at a holding potential of −70 mV and 0 mV, respectively, in voltage-clamp configuration. Recording pipettes were pulled from borosilicate glass (open tip resistance 3–5 MΩ; King Precision Glass Co. Claremont, CA). The pipette recording solution contained (in mM) 140 Cs-gluconate, 4 CsCl, (or 126 K-gluconate with 4 KCl) 10 HEPES, 4 MgATP, 0.3 NaGTP, and 10 Phosphocreatine; pH was adjusted to 7.2 with CsOH or KOH with an osmolarity of 290–295 mOsm. Electrophysiological recordings were performed using a Multiclamp 700B amplifier (Molecular Devices), low pass filtered at 3 kHz using a Bessel filter and digitized at 20 kHz with Digidata 1440A analog-digital interface (Molecular Devices). Series resistance was <25 MΩ and was monitored periodically during the recordings. Recordings were discontinued if series resistance changed by more than 20% during the recording or reached 25 MΩ. Electrophysiological traces were analyzed using Mini Analysis (version 6.0.7, Synaptosoft Inc.). Frequency and amplitude of sEPSCs and sIPSCs were detected and included if the amplitude of the synaptic current was greater than three times root mean square noise level using Mini Analysis.

### Hippocampal DGC dispersion

After electrophysiology recordings, coronal hippocampal slices were placed in 4% paraformaldehyde in phosphate-buffered saline (PBS). Slices were rinsed in PBS then equilibrated in 30% sucrose solution in PBS prior to cryo-sectioning. Slices were re-sectioned with a thickness of 30 μM and mounted on Superfrost microscope slides with Vectashield mounting medium containing DAPI. Images of the dorsal dentate gyrus were acquired using a SPOT RT-Slider camera (Diagnostic Instruments, Sterling Heights, MI) on an upright microscope (BX43, Olympus) using SPOT Advanced Software (Diagnostic Instruments). Hippocampal DGC dispersion was quantified using ImageJ software. Dispersion of granule cells was defined as being at least two times the average linear micron distance of the vehicle DGC suprapyramidal blade measured from the hilar border to the outer margin of the DGC layer (Moura et al., 2019). Dispersion was only detected in mice that developed spontaneous recurrent seizures.

### Statistical analysis

Statistical measures were performed with Prism (GraphPad, San Diego, CA). Data were disaggregated by sex; no sex-dependent differences were detected for any measure, so sexes were combined for all analyses. Statistics of sex-dependent differences are shown in Supplemental Table 1 using chi-square test for seizure distribution and unpaired-tests for electrophysiology measurements comparing males and females within each genotype and treatment. Data were tested for normality by a Shapiro–Wilk test and non-parametric tests were used where appropriate. Seizure analysis (i.e., seizure number, duration, latency, and spiking frequency) was compared using an unpaired *t*-test between genotypes. Distribution analysis of spontaneous seizure development was compared using chi-square analysis. Electrophysiological data were analyzed using two-way ANOVA with Tukey test for multiple comparisons. Data are presented as mean ± SEM and statistical significance was set to *p* < 0.05 for all tests. See Supplemental Table 2 for description of statistical analyses for all figures.

## Results

### A lower percentage of mice lacking tau expression developed SE with fewer seizures during the induction period

**Figure 1:**
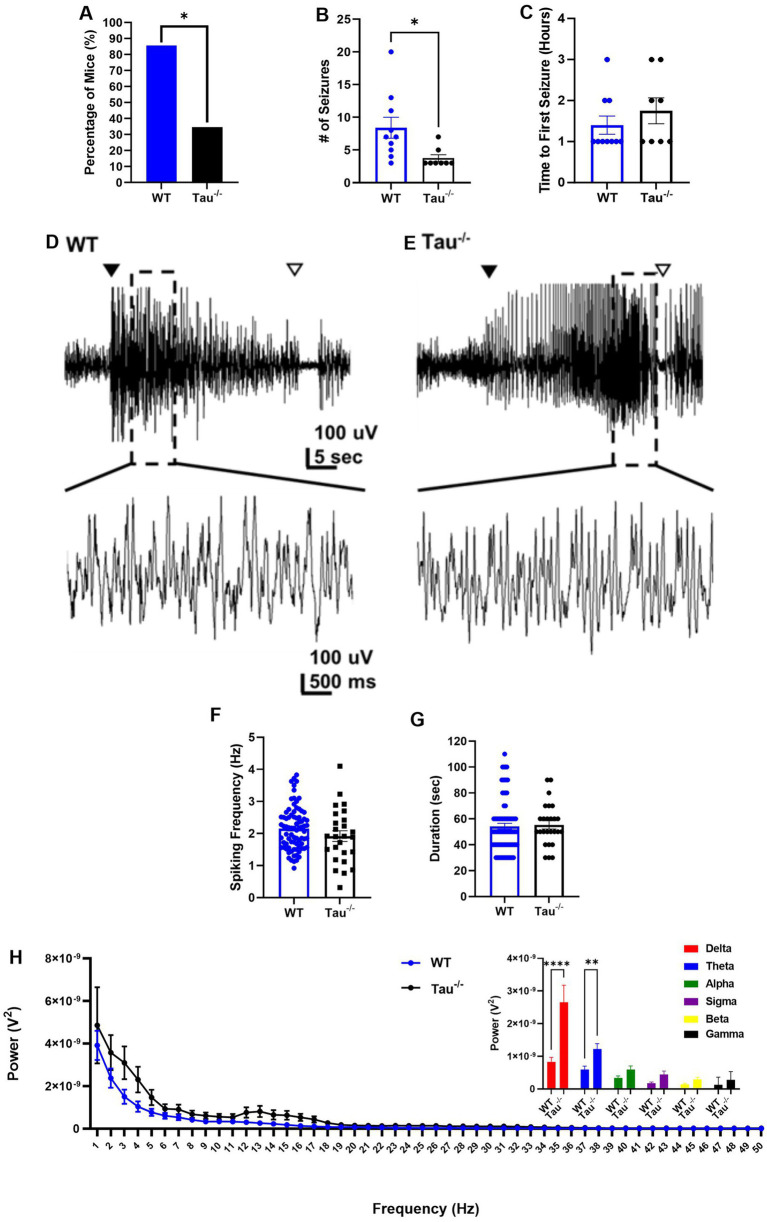
Tau^−/−^ mice are less likely to exhibit SE after intrahippocampal kainate (IHK) injection and if SE develops, mice exhibit fewer convulsive seizures compared to wildtype controls. **(A)** Percentage of wildtype (WT) control (42/49) and tau^−/−^ mice (17/44) that developed SE after kainate injection; *p < 0.01, chi-square test. **(B)** Number of seizures from mice that exhibited SE defined by electroencephalography (EEG) and behavior (WT *N* = 10, tau^−/−^ *N* = 8; *p < 0.05, unpaired *t*-test) between the time of recovery from anesthesia and 4 h post-recovery. Each point represents the number of seizures from a mouse from either genotype. **(C)** Time to first convulsive seizure after recovery from mice fitted with EEG (WT *N* = 10, tau^−/-^*N* = 8; *p* > 0.05; unpaired *t*-test) **(D)** Representative EEG traces of convulsive seizures from WT control and **(E)** tau^−/−^ mice during SE. Closed arrow indicates the start of the behavioral/electrographic seizure and open arrow indicates the end. Temporally expanded sections of each trace are shown below. **(F)** Epileptiform activity quantified as spiking frequency (i.e., spikes per second) in convulsive seizures during SE. Each point represents a seizure from either genotype (*p* > 0.05, unpaired *t*-test). **(G)** Duration of epileptiform activity of each convulsive seizure during the induction period after IHK injection from either genotype (WT *N* = 10, tau^−/−^ *N* = 8, *p* > 0.05, unpaired *t*-test). **(H)** Periodogram of electroencephalography signal during convulsive seizures from both genotypes ranging from 0.5 Hz to 50 Hz. Both genotypes exhibited a frequency peak at 1 Hz indicating dominant frequency range within the delta frequency band. No significant difference detected in total power calculated as area under the curve (AUC; *p* = 0.0749; unpaired *t*-test). Inset is summed power within each frequency band. Tau^−/−^ mice exhibit greater power during SE seizures within the delta and theta frequency bands compared to wildtype mice (****p < 0.0001; **p < 0.01). Frequency bands were defined as: delta: 0.5–4 Hz; theta: 4–8 Hz; alpha: 8–12 Hz; sigma: 12–16 Hz; beta: 16–30 Hz; gamma: 30–100 Hz.

Intrahippocampal kainate-induced SE leads to the development of spontaneous seizures after a latent period, where convulsive seizures during SE reliably result in TLE (Bouilleret et al., 1999; Kang et al., 2021; Rusina et al., 2021). We previously determined that at least three convulsive seizures (Racine seizure scale 3–5; Racine, 1972) after intraperitoneal pilocarpine injection predicted epileptogenesis in C57BL/6 J and other mouse strains (Shibley and Smith, 2002; Winokur et al., 2004; Bhaskaran and Smith, 2010), so emergence of multiple convulsive seizures after IHK was defined as status epilepticus (SE) that was sufficient to trigger TLE. Unilateral IHK injection in the dorsal hippocampus results in SE in mice starting ~1–2 h after the injection; wildtype C57BL/6 J mice reliably develop convulsive SE after IHK (Welzel et al., 2020; Kang et al., 2021). Injection of kainate resulted in 85.71% of wildtype controls (42/49 mice) and 34.69% of tau^−/−^ (17/46 mice) exhibiting convulsive SE during the induction period (*p* < 0.05; Figure 1A). Mice that were not characterized as exhibiting convulsive SE did not develop convulsive seizures at any point during the induction period but demonstrated epileptiform spiking in EEG recordings, which has been reported by others (Haussler et al., 2012). Tau^−/−^ mice that exhibited convulsive SE during the induction period had fewer convulsive (i.e., Racine 3–5) and electrographic seizures compared to wildtype controls (*p* < 0.05; Figure 1B). Further there was no difference in the latency to the first convulsive seizure between tau^−/−^ (84 ± 14.37 min) and wildtype controls (82 ± 7.98 min) (*p* = 0.36, unpaired-test; Figure 1C), with majority of animals experiencing the first convulsive seizure 1–2 h after recovery from anesthesia. Overall, the results suggest that susceptibility to SE is lower in tau^−/−^ mice, consistent with results of previous studies showing reduced seizure activity in mice lacking tau expression in models of genetic epilepsies and lower seizure susceptibility to chemo-convulsant evoked seizures (Roberson et al., 2007; DeVos et al., 2013; Gheyara et al., 2014; Li et al., 2014; Putra et al., 2020).

To determine potential differences in the severity of convulsive seizures during SE, we assessed epileptiform spiking frequency and power spectral density during the ictal phase of seizures and the duration of the epileptiform activity (Figures 1D–G). After injection of kainate or saline, a subset of mice (IHK: wildtype *N* = 18, tau^−/−^ *N* = 15; vehicle: wildtype *N* = 7, tau^−/−^ *N* = 9) were fitted with a 4-channel surface EEG connector with one channel screw implanted near the injection burr hole and recorded as described in the methods section. Electrographic seizures were assessed during behavioral seizures described in the methods. There were no significant differences detected in spiking frequency (*p* > 0.05) or electrographic seizure duration (*p* > 0.05) between tau^−/−^ or wildtype mice (Figures 1D–G). We further analyzed spectral analysis of convulsive seizures during SE from wildtype and tau^−/−^ animals. The periodogram demonstrated no significant difference in the total power or any difference in the peak at specific frequencies, with convulsive seizures from both wildtype and tau^−/−^ mice exhibiting the dominant signal frequency within the delta band frequency range, suggesting similar signal strength and oscillatory pattern during SE (*p* > 0.05, unpaired *t*-test; Figure 1H). Interestingly the summed power across specific frequency bands of seizures demonstrated a significant increase in power in the delta and theta power bands in tau^−/−^ animals compared to wildtype animals, suggesting that while the signal strength does not differ, the power has shifted to a more hypersynchronous signal activity (*p* < 0.05, 2-way ANOVA; Figure 1H inset). Thus, whereas fewer tau^−/−^ mice expressed SE after IHK, with a lower convulsive seizure frequency, tau deletion did not overtly alter spiking frequency, electrographic activity, or mean seizure duration but may alter cortical network activity of individual seizures during SE.

### Development of TLE in tau^−/−^ mice

Emergence of multiple convulsive seizures after IHK (i.e., SE) is considered to be sufficient to trigger TLE development in C57BL/6 J mice (Kang et al., 2021; Rusina et al., 2021), similar to systemic chemo-convulsant treatments to induce TLE development (Shibley and Smith, 2002). It is possible that the lower severity of SE in tau^−/−^ mice predicts a lower proportion of those mice developing spontaneous seizures. However, studies examining tau reduction or deletion have not described whether resistance to chemoconvulsant-induced SE alters spontaneous seizure development independent of other pathologies in a model of acquired TLE (Roberson et al., 2007; Roberson et al., 2011; DeVos et al., 2013).

**Figure 2:**
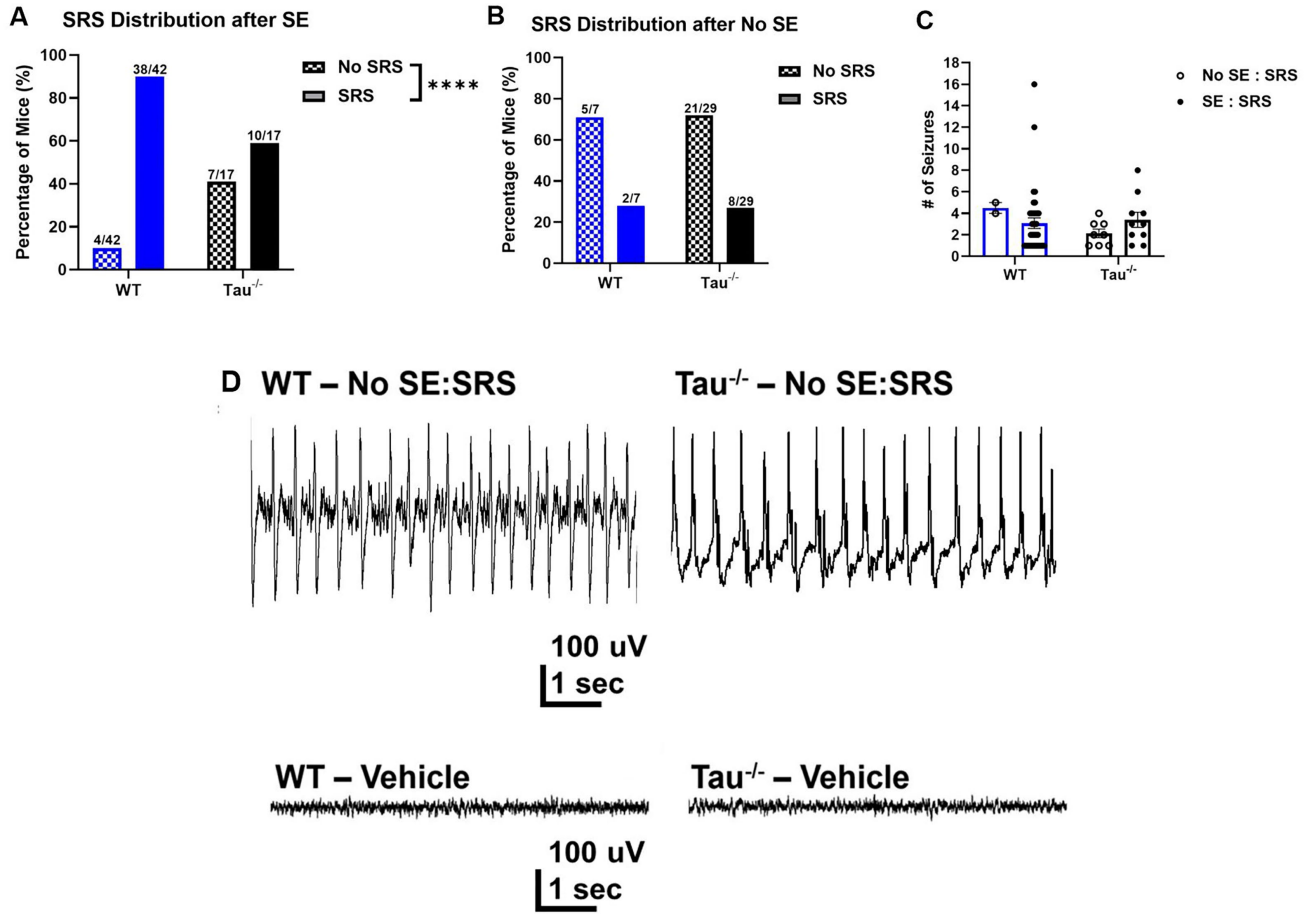
Tau^−/−^ mice develop spontaneous seizures after SE, but at a lower rate. **(A)** Percentage of WT control and tau^−/−^ mice that developed spontaneous seizures after exhibiting SE (WT *N* = 42; tau^−/−^ *N* = 17; ****p < 0.0001, chi-square test). After experiencing convulsive SE, fewer tau^−/−^ mice developed SRS compared to wildtype controls. **(B)** Percentage of wildtype controls and tau^−/−^ mice that developed spontaneous seizures without exhibiting convulsive SE (WT *N* = 7; tau^−/−^ *N* = 29; *p* > 0.05, chi-square test). There was no difference in the percentage of mice of either genotype that developed SRS without convulsive SE. **(C)** Number of spontaneous recurrent seizures per 72 h from mice defined by electroencephalography and behavior. Each point represents the number of seizures from a mouse of either genotype. No difference was detected across genotypes or SE expression. **(D)** Representative EEG traces of epileptiform spiking (upper traces) shortly after IHK treatment from mice that did not develop convulsive seizures after IHK injection but developed spontaneous seizures after a latent period (No SE: SRS). Representative EEG traces from vehicle-treated mice are also shown (lower traces).

Using video and video-EEG, we investigated whether lack of tau expression alters chronic seizure expression in TLE by examining the percentage of mice that exhibited SRS 3 weeks after IHK. Of the animals that exhibited SE during the induction period and developed spontaneous seizures (i.e., SE: SRS), a significantly smaller proportion of only 10/17 (58.82%) tau^−/−^ animals exhibited both while 38/42 (90.47%) wildtype mice exhibited both convulsive SE and developed SRS (Figure 2A; *p* < 0.05). Chi-square analysis of the distribution of animals that developed SRS after exhibiting SE, suggests that there is an associative effect of tau expression on TLE epileptogenesis after SE.

Interestingly, spontaneous seizures developed in subsets of wildtype and tau^−/−^ mice that did not exhibit convulsive SE (i.e., No SE: SRS) (Figure 2B). In the subset of mice that did not exhibit convulsive SE, there was a similar proportion of wildtype and tau^−/−^ mice that developed spontaneous seizures (wildtype: 2/7, 28.57%; tau^−/−^: 8/29, 27.59%; *p* > 0.05). Chi-square analysis revealed that there was no effect of tau expression on SRS development when convulsive SE was not expressed after the IHK injection. Further, the number of spontaneous seizures did not significantly differ between the groups that did or did not exhibit convulsive SE but developed SRS (*p* > 0.05; Figure 2C). Overall, these results demonstrate that mice lacking tau expression develop acquired TLE in the IHK model, which has not been shown previously.

In mice of both genotypes (i.e., wildtype and tau^−/−^) that did not experience SE after IHK injection, EEG traces during the induction period displayed epileptiform spiking after kainate administration (Figure 2D), suggesting that nonconvulsive epileptiform activity caused by the IHK injection may be sufficient to trigger or positively influence the epileptogenic process in mice. Due to the low number of electroencephalographically recorded wildtype mice (*N* = 1) that failed to develop SE but went on to develop SRS compared to tau^−/−^ mice (*N* = 2), quantitative comparison of epileptiform spiking between the two genotypes was not possible. Even so, the epileptiform spike frequency during the induction period of tau^−/−^ mice in the No SE/SRS group (1.331 ± 0.552) was not obviously different from the wildtype mouse (1.119). It is possible differences may not have been observed due to a small cohort of animals.

**Figure 3:**
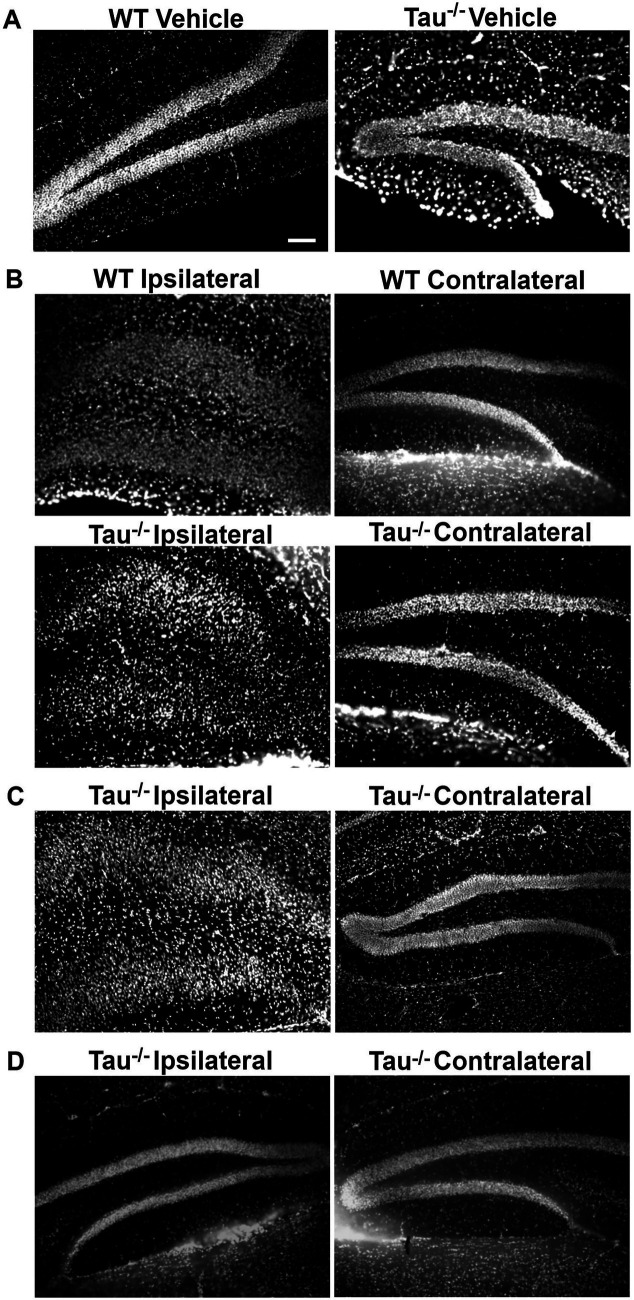
Hippocampal dispersion in tau^−/−^ mice is dependent on SRS development regardless of convulsive SE. Representative images of DAPI staining in the dentate gyrus. **(A)** Vehicle treated wildtype and tau^−/−^ mice. **(B)** Wildtype and tau^−/−^ mice that exhibited SE and later developed SRS demonstrating dispersion of granule cell layer ipsilateral to IHK (SE: SRS). **(C)** Tau^−/−^ mouse that did not exhibit SE but developed SRS (No SE: SRS) demonstrating dispersion of the granule cell layer ipsilateral to IHK. **(D)** IHK treated Tau^−/−^ mouse that did not exhibit SE or develop SRS (No SE: No SRS). No dispersion observed ipsilateral to IHK injection. Scale bar = 50 μM.

Hippocampal sclerosis and dispersion of DGCs have been described in the IHK model of TLE (Suzuki et al., 1995; Bouilleret et al., 1999; Riban et al., 2002; Marx et al., 2013) and may result from kainate toxicity or SE, or it may be an emergent property of epileptogenesis. Mice of both genotypes that exhibited convulsive SE and developed SRS (i.e., SE: SRS) demonstrated DGC dispersion ipsilateral, but not contralateral, to IHK injection, consistent with previous reports of TLE development in IHK treated mice (Bouilleret et al., 2000) (Figures 3A,B). We measured the distance of the observed dispersion, as described in the Methods. Intriguingly, the subset of mice that did not exhibit convulsive seizures during SE but nonetheless developed SRS (i.e., No SE: SRS) also exhibited granule cell dispersion ipsilateral to IHK injection that was similar to that observed in mice that experienced convulsive SE and SRS (Figures 3B,C). Mice that failed to develop SRS (i.e., No SE: No SRS and SE: No SRS) did not develop DGC dispersion (Figure 3D). Ipsilateral DGC dispersion in tau^−/−^ mice that developed SRS after SE (SE: SRS) (198.21 ± 23.01, *N* = 3) was not numerically different from wildtype mice (WT: 195.89 ± 23.69, *N* = 5; *p* > 0.05, unpaired-test). On the contralateral hemisphere where dispersion was not observed in any genotype that developed SRS after SE, the width of the granule cell layer was also not numerically different between wildtype (71.34 ± 8.05, *N* = 5) and tau^−/−^ mice (66.06 ± 10.61, *N* = 3) *p* > 0.05, unpaired *t*-test, nor was it different from vehicle treated animals (77.69 ± 4.23, *N* = 4, unpaired *t*-test). Due to the low percentage of wildtype mice that failed to develop convulsive SE or SRS, quantitative analysis with comparison to tau^−/−^ animals was not possible. Even so, we did not observe differences in hippocampal dispersion between tau^−/−^ mice that did not exhibit SE but developed SRS (No SE: SRS; 219.38 μM; *N* = 1) and those that did exhibit SE and develop SRS (SE: SRS). Together, these findings suggest that dispersion of the granule cell layer is an outcome of TLE development rather than kainate toxicity or SE alone.

Overall, lack of tau expression reduces the likelihood of TLE development, but it does not prevent epileptogenesis in mice after IHK injection. Additionally, tau expression did not affect TLE development in mice that did not experience SE, suggesting that epileptogenesis itself is not dependent on tau expression and neither is it entirely dependent on convulsive SE.

### Spontaneous seizures in tau^−/−^ mice that develop TLE are less severe

**Figure 4:**
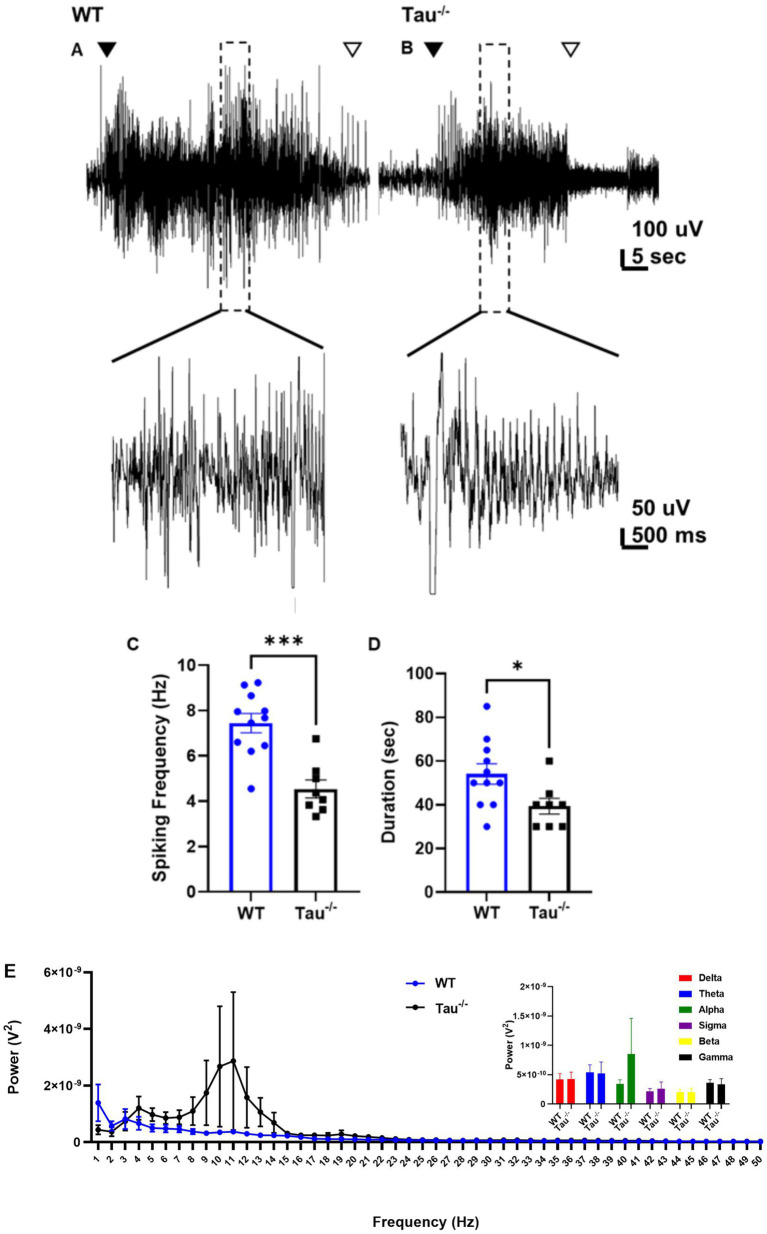
Spontaneous seizures in tau^−/−^ exhibit lower spike frequency and are shorter in duration. **(A)** Representative EEG traces of spontaneous seizures from WT control and **(B)** tau^−/−^ mice 3 weeks after IHK injection. The closed arrow indicates the start of the seizure, and the open arrow indicates the end. Expanded sections of traces are shown at a higher magnitude below. **(C)** Epileptiform activity is quantified as spiking frequency (i.e., spikes per second) during the ictal portion of spontaneous seizures. Each point represents a convulsive seizure from either genotype. Spike frequency during spontaneous seizures from tau^−/−^ mice was lower than in WT mice (***p < 0.001, unpaired *t*-test). **(D)** Duration of epileptiform activity during each spontaneous seizure from either genotype. Spontaneous seizures in tau^−/−^ mice were shorter compared to wildtype controls (* < 0.05, unpaired *t*-test). **(E)** Periodogram of EEG signal during spontaneous convulsive seizures after development of TLE from both genotypes ranging from 0.5 Hz to 50 Hz. Tau^−/−^ genotypes exhibited a frequency peak in at 1 Hz suggesting a dominant frequency range within the delta frequency band. Total power calculated as area under the curve (AUC) suggests greater total power during convulsive seizures from tau^−/−^ mice (*p < 0.05; unpaired *t*-test). Inset is summed power within each frequency band. No significant difference was detected across the frequency bands between wildtype and tau^−/−^ mice (*p* > 0.05). Frequency bands were defined as: delta: 0.5–4 Hz; theta: 4–8 Hz; alpha: 8–12 Hz; sigma: 12–16 Hz; beta: 16–30 Hz; gamma: 30–100 Hz.

Since we found that tau^−/−^ mice develop SRS at a lower rate than wildtype controls, we investigated how lack of tau expression altered electrographic seizures once TLE developed (Figure 4). Spontaneous electrographic seizures in tau^−/−^ mice that developed TLE exhibited lower epileptiform spiking frequency during the ictal phase (*p* < 0.05) and the duration of the seizures was significantly shorter compared to wildtype mice (*p* < 0.05; Figures 4C,D). We found no difference in spike frequency or duration of spontaneous seizures between tau^−/−^ mice that did not exhibit SE but developed SRS (i.e., No SE: SRS) (spike frequency: 4.19 ± 0.35; duration: 30 ± 0.0, *N* = 2) and those that exhibited both (i.e., SE: SRS) (spike frequency: 4.88 ± 0.73; duration: 35 ± 2.89, *N* = 2) (*p* > 0.05). Therefore, we included spike frequency from both groups of tau^−/−^ mice in Figure 4. Spectral analysis of spontaneous seizures from both genotypes revealed an overall greater total power contribution in tau^−/−^ mice compared to wildtype (*p* < 0.05; Figure 4E). Interestingly, there was a shift in the dominant frequency in tau^−/−^ mice to the alpha power frequency band (i.e., 8–12 Hz) compared to wildtype mice whose seizures peaked in the delta range (i.e., 0.5–4 Hz). It should be noted that the large variation in the dominant frequency shift to alpha power can be attributed to seizures from one tau^−/−^ animal. Even so, there was no significant difference detected in the alpha power band (*p* > 0.05; Figure 4E inset). Overall, spectral analysis indicates that underlying seizure dynamics are modified in tau^−/−^ mice after development of TLE. Quantitative analysis comparing spike frequency of spontaneous seizures in mice that did not exhibit SE (No SE: SRS) between genotypes was not possible due to only one wildtype animal exhibiting this seizure phenotype out of the 16 recorded using EEG. Additionally, of the 49 IHK-treated wildtype mice, only 2 developed spontaneous seizures without exhibiting convulsive SE after IHK injection.

Thus, after IHK, mice lacking tau expression were less likely to develop TLE and if they did, their spontaneous seizures were less severe, suggesting tau protein may facilitate epileptogenesis-associated neuroplasticity, increasing the severity of individual spontaneous unprovoked seizures. Therefore, lack of tau expression is somewhat protective against spontaneous seizure development if convulsive SE is reached after IHK and if mice lacking tau expression develop TLE their seizures are less severe, but they exhibit altered ictal oscillatory patterns. Further, SE expression has little effect on spontaneous seizure burden once TLE develops. Together, this highlights a significant role of tau protein expression on evoked seizure generation after IHK, which impacts acquired TLE epileptogenesis, and that tau expression also plays a role in unprovoked spontaneous seizures once TLE has developed.

### After development of TLE, excitatory synaptic input to DGCs increases in all mice regardless of tau expression

**Figure 5:**
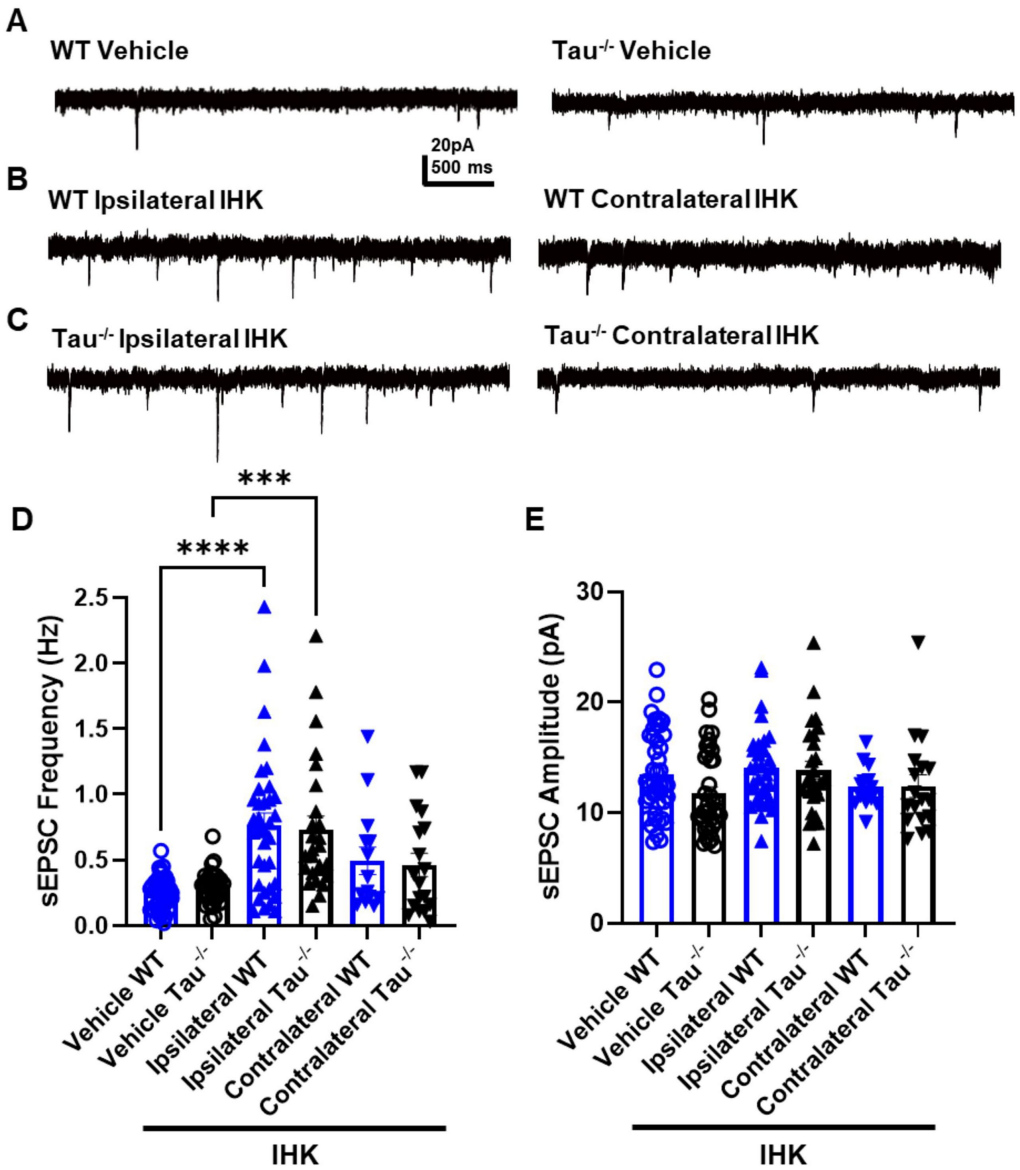
Excitatory synaptic input to DGCs is increased ipsilateral to IHK in all mice that developed TLE. **(A)** Representative sEPSC traces recorded in DGCs from vehicle-treated wildtype (WT) control and tau^−/−^ mice. **(B)** Representative sEPSC traces from wildtype mice ipsilateral (left) and contralateral (right) to IHK injection. **(C)** Representative sEPSC traces from IHK-treated tau^−/−^ mice. **(D)** Frequency of sEPSCs from sham and IHK-treated mice. Each point represents the frequency of events from one cell. After development of TLE, sEPSC frequency is increased similarly in both genotypes ipsilateral to IHK injection. **(E)** Amplitude of sEPSCs from sham and IHK-treated mice. Each point represents the average event amplitude from one cell; no significant differences were detected in any group. WT: sham *N* = 11, *n* = 46; IHK = 12, Ipsilateral *n* = 32, Contralateral *n* = 15. Tau^−/−^: sham *N* = 7, *n* = 37; IHK *N* = 8, Ipsilateral *n* = 26, Contralateral *n* = 18). ****p < 0.0001, ***p < 0.001, *p < 0.05, Two-way ANOVA.

We hypothesized that tau expression was involved in the synaptic reorganization associated with the development of spontaneous seizures, where lack of expression suppresses the synaptic reorganization in DGCs that occurs during the development of TLE. Formation of new recurrent excitatory synaptic connections among DGCs has been shown to be associated with acquired TLE development in rodents (Cronin et al., 1992; Buckmaster and Dudek, 1997; Bouilleret et al., 1999; Winokur et al., 2004; Hunt et al., 2009). As an assay of overall excitatory synaptic input, we examined the frequency of sEPSCs in DGCs from tau^−/−^ and wildtype control mice that developed TLE, confirmed by behavioral and electrographic seizure monitoring (Figure 5). In all mice that developed TLE after IHK, significantly increased sEPSC frequency was observed in DGCs ipsilateral to the injection site compared to vehicle-treated controls, regardless of tau expression (*p* < 0.05; Figure 5D, Table 1). There was no significant difference in sEPSC frequency between DGCs from vehicle treated mice and those contralateral to IHK injection (*p* > 0.05), suggesting development of TLE is not associated with an increase in excitatory synaptic input to DGCs contralateral to IHK. Further we found no detectable difference between sEPSC frequency in DGCs from tau^−/−^ or wildtype mice that developed TLE ipsilateral (*p* > 0.05) or contralateral (*p* > 0.05) to the IHK injection; sEPSC frequency was also not affected by genotype in vehicle treated mice (*p* > 0.05). In addition, there was no difference in sEPSC frequency between vehicle- and IHK-injected mice of either genotype that did not develop SRS (*p* > 0.05), suggesting that the increased excitatory synaptic input to DGCs ipsilateral to IHK injection is associated with TLE development rather than kainate treatment or SE alone. There was also no difference detected in the amplitude of sEPSCs events between vehicle- and IHK-injected mice of either genotype (*p* > 0.05; Figure 5E, Table 1).

**Table 1:** Summary of electrophysiology measurements.

Genotype and treatment			Point Estimate ± SEM (n)			
			sEPSC Frequency (Hz)	sEPSC Amplitude (Hz)	sIPSC Frequency (Hz)	sIPSC Amplitude (Hz)
Wildtype (Ipsilateral and Contralateral)		Veh	0.2405 ± 0.0188 (44)	13.496 ± 0.5765 (44)	0.8611 ± 0.0063 (35)	24.081 ± 1.5300 (35)
Tau^-/-^ (Ipsilateral and Contralateral)		Veh	0.2897 ± 0.0199 (37)	11.7616 ± 0.6059 (37)	0.4179 ± 0.0375 (29)*	28.3152 ± 1.9804 (29)
Wildtype	Ipsilateral	IHK	0.7686 ± 0.0894 (35)*	14.029 ± 0.6327 (35)	0.0819 ± 0.0149 (16)* ^**#**^	34.5543 ± 0.0149 (16)
	Contralateral	IHK	0.4943 ± 0.1051 (14)	12.34 ± 0.4991 (14)	0.6153 ± 0.0672 (15) ^+^ *	29.3133 ± 3.6616 (15)
Tau^-/-^	Ipsilateral	IHK	0.7346 ± 0.0997 (26)**	13.8929 ± 0.8209 (26)	0.1576 ± 0.0354 (17)** ^**#**^	36.8018 ± 1.9492 (17)
	Contralateral	IHK	0.4622 ± 0.0891 (18)	12.412 ± 1.0146 (18)	0.8536 ± 0.1124 (19) ^+^ **	32.9285 ± 2.5330 (19)

^*^different from vehicle wildtype; **different from vehicle tau−/−; ^+^different from ipsilateral same genotype; ^#^different from contralateral same genotype.

Together these results suggest that tau protein likely does not affect the excitatory synaptic reorganization in the local dentate gyrus circuit that is associated with TLE development. Therefore, the differential effects of tau deletion on evoked and spontaneous seizures in the IHK model are likely to be a result of other circuit reorganization that may modulate seizure initiation and epileptogenesis.

### DGCs receive less inhibitory synaptic input in tau^−/−^ mice, but development of TLE increases contralateral inhibition in mice lacking tau expression

**Figure 6:**
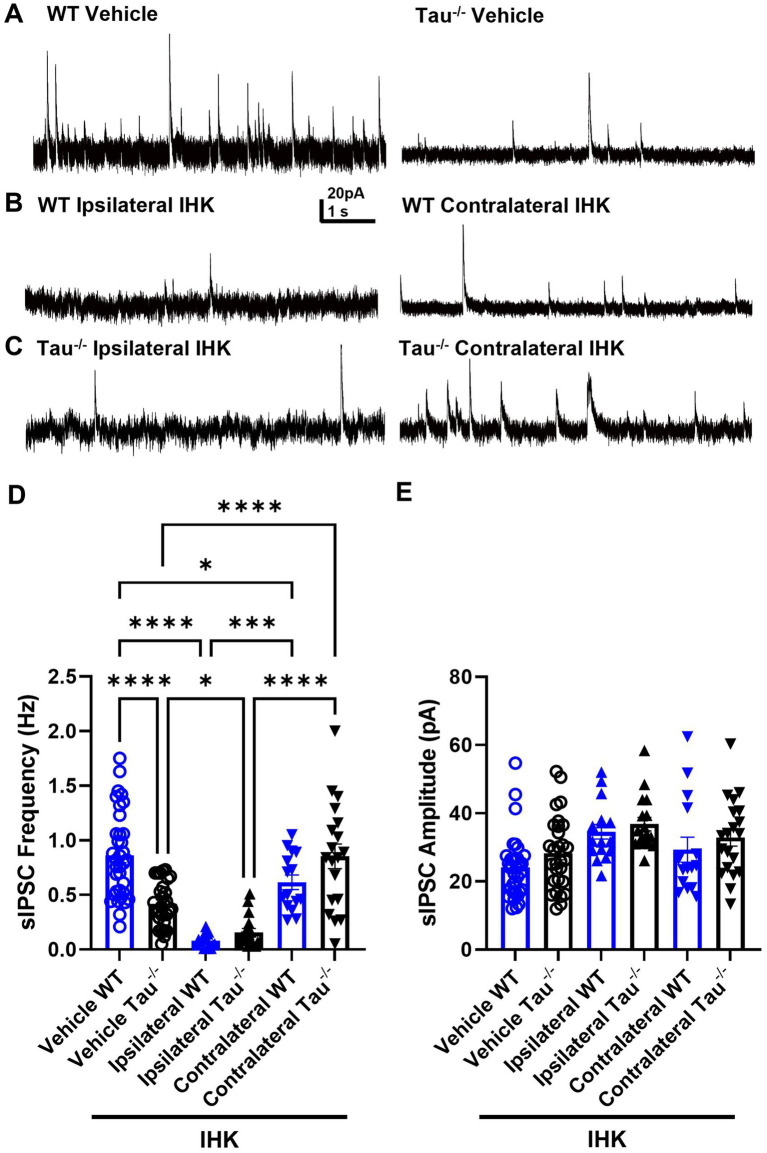
DGCs receive less inhibitory synaptic input in naïve tau^−/−^ mice and epileptogenesis decreases sIPSC frequency in both genotypes ipsilateral to IHK but increases contralateral inhibition in tau^−/−^ mice. **(A)** Representative sIPSC traces recorded in DGCs from vehicle-treated wildtype (WT) control and tau^−/−^ mice. **(B)** Representative sIPSC traces from IHK-treated wildtype mice that developed TLE. **(C)** Representative sIPSC traces from IHK-treated tau^−/−^ mice that developed TLE. **(D)** Frequency of sIPSCs from sham and IHK-treated wildtype mice. Each point represents the frequency of events from one cell. sIPSC frequency is lower in vehicle tau^−/−^ mice compared to vehicle WT (*p* < 0.05). sIPSC frequency is reduced ipsilateral to IHK injection in both tau^−/−^ and wildtype mice compared to their vehicle controls (*p* < 0.05). sIPSC frequency is reduced in IHK- treated wildtype mice contralateral to IHK compared to vehicle-treated wildtype mice (*p* < 0.05). sIPSC frequency is increased contralateral to IHK injection in tau^−/−^ mice compared to vehicle-treated tau^−/−^ mice and to sIPSC frequency in the ipsilateral hemisphere after IHK (*p* < 0.05). **(E)** Amplitude of sEPSCs from sham and IHK-treated mice. Each point represents the average event amplitude from one cell. No difference detected in amplitude across genotypes or treatments (*p* > 0.05). WT: sham *N* = 8, *n* = 35; IHK = 5, Ipsilateral *n* = 18, Contralateral *n* = 15. Tau^−/−:^ sham *N* = 8, *n* = 29; IHK *N* = 6, Ipsilateral *n* = 19, Contralateral *n* = 20). ****p < 0.0001, **p < 0.01, *p < 0.05, Two-way ANOVA.

The development of spontaneous seizures is often accompanied by interneuron loss, especially ipsilateral to IHK injection, which may contribute to hippocampal network excitability (Kobayashi and Buckmaster, 2003). We assessed the possibility that SRS development in tau^−/−^ mice is associated with preservation of inhibitory synaptic input to DGCs by recording sIPSCs in DGCs from mice that developed spontaneous seizures (Figure 6, Table 1). Unsurprisingly, inhibitory synaptic input was reduced ipsilateral to IHK injection in both tau^−/−^ and wildtype mice compared to vehicle treated controls (*p* < 0.05; Figure 6D, Table 1), similar to previous reports in TLE models (Morin et al., 1998; Kobayashi and Buckmaster, 2003). There was no difference in event frequency between IHK-treated tau^−/−^ and wildtype mice in DGCs after development of TLE (*p* > 0.05). Interestingly, tau^−/−^ mice that developed TLE exhibited significantly increased sIPSC frequency in DGCs contralateral to IHK injection compared to vehicle-treated tau^−/−^ mice and ipsilateral to the IHK injection (*p* < 0.05; Figure 6D, Table 1), whereas sIPSC frequency in contralateral DGCs from IHK-treated wildtype mice was reduced compared to vehicle-treated wildtype mice (*p* < 0.05). Together this suggests that after tau^−/−^ mice develop TLE, DGCs contralateral to the IHK lesion receive more inhibitory synaptic input, which may contribute to the lower spontaneous seizure burden in these mice. Interestingly, DGCs from vehicle-treated tau^−/−^ mice normally receive less inhibitory synaptic input compared to wildtype controls, since sIPSC frequency is significantly lower in vehicle-treated tau^−/−^ mice (*p* < 0.05; Figure 6D; Table 1), which has not been shown before. No difference was detected in the average amplitude of sIPSC events from DGCs across genotype or treatment (*p* > 0.05; Figure 6E, Table 1). Thus, tau likely plays a role in spontaneous inhibitory synaptic transmission within the dentate gyrus and its expression influences the inhibitory synaptic reorganization that occurs during TLE development, which may play a role in the modified seizure expression in tau^−/−^ mice.

## Discussion

The results in this study demonstrate that lack of tau expression does not prevent evoked seizures or the subsequent development of TLE using the IHK model, which has not been previously shown. Rather, deletion of tau elicits differential effects on IHK-evoked SE and epileptogenesis. Mice lacking tau expression were less likely to exhibit convulsive SE after IHK injection, consistent with other studies on the effects of tau deletion or reduction on evoked seizures (DeVos et al., 2013; Li et al., 2014; Putra et al., 2020; Shao et al., 2022). Whereas genetic deletion of tau influenced evoked seizure initiation, it did not quell convulsive seizures once SE developed nor did it prevent the development of TLE. To date there have been limited studies investigating how lack of tau expression influences epileptogenesis in mouse models of acquired TLE. Canonically, SE triggers the process of epileptogenesis in chemoconvulsant models of TLE (Riban et al., 2002). While tau^−/−^ mice developed TLE at a lower rate after experiencing convulsive SE, a subset of mice from both genotypes developed SRS without exhibiting convulsive SE after IHK injection and the rate at which tau^−/−^ mice developed TLE in the absence of IHK induced SE was virtually identical to that in wildtype mice. This suggests the influence of tau expression on TLE development is mainly related to components of the epileptogenic process that are related to SE severity and not the process of epileptogenesis itself. A mouse model of post-traumatic epilepsy (i.e., CCI) often triggers the process of epileptogenesis independent of SE (Hunt et al., 2009; Hunt et al., 2010; Kang et al., 2022). Another chemoconvulsant-SE model (i.e., intraperitoneal kainate treatment) results in varying severity of SE (Puttachary et al., 2015), but the mechanisms of how a brain insult like SE contributes to TLE development remain unclear. Our results suggest that nonconvulsive seizures or epileptiform spiking may be sufficient to trigger the development of spontaneous seizures in the IHK model in both genotypes, and tau deletion did not impede the epileptogenic process.

Pathological murine models of Alzheimer’s Disease (i.e., tauopathies and/or amyloid beta) exhibit impairments in synaptic plasticity and memory information processing (Chung et al., 2020). Here, we investigated whether mice lacking tau expression exhibited altered epileptiform activity during convulsive seizures after IHK injection (i.e., during SE) or after TLE developed (i.e., during SRS). Although tau^−/−^ mice exhibited reduced seizure expression during SE, the seizure duration and epileptiform spike frequency of convulsive seizures were not different from wildtype controls. Even so, spectral analysis revealed increased power in the delta and theta frequency bands, suggesting increased neural activity of specific circuit level recruitment. To wit, theta oscillation is associated with the function of both parvalbumin and somatostatin expressing interneurons with activation of these subtypes influencing theta rhythm in the hippocampus (Amilhon et al., 2015; Maheshwari, 2020). Unlike seizures during SE, spontaneous seizures that occurred after TLE developed in tau^−/−^ mice exhibited lower spike frequency and were shorter in duration than in wildtype controls. Interestingly, spectral analysis of spontaneous seizures in tau^−/−^ mice exhibited greater total power and peak frequency shift to the theta/alpha range. Together, this suggests that although the duration and mean spike frequency during the ictal phase is less, the overall signal strength is higher as compared to wildtype controls. It is likely that seizure dynamics are modified once TLE develops in mice lacking tau expression, with a more synchronized neural network with greater oscillatory power between the theta and alpha frequencies. Mechanistically, convulsive seizures after IHK injection in mice lacking tau expression may therefore involve activity of cell types responsible for generating frequencies that may be acting in an anti-epileptogenic manner and further contributing to modified epileptogenesis. Therefore, tau deletion affects seizures in a manner that is consistent with a mechanistic effect on neuronal excitability, but effects of tau deletion on epileptogenesis *per se* were not as apparent.

Tau deficient mice have previously been shown to exhibit slower theta oscillations in the hippocampus and reduced gamma synchronization in the neocortex and hippocampus (Cantero et al., 2011). Distinct populations of interneurons are thought to contribute to hippocampal and neocortical theta and gamma oscillations (Sohal et al., 2009; Lisman and Jensen, 2013; Chung et al., 2020). Dysfunctions in interneuron populations could underlie impaired oscillations in models of AD where reduced power in lower frequency bands corresponds with reduced inhibitory synaptic signaling to excitatory pyramidal cells (Chung et al., 2020), and interneuron circuit function is altered after TLE development in rodents (Buckmaster and Dudek, 1997; Buckmaster and Wen, 2011). Studies examining brain oscillations commonly analyze local field potentials in specific regions (e.g., hippocampus and/or necortex) that allows for elucidating the generation of oscillation frequencies from specific cell types (Lisman and Jensen, 2013; Pernia-Andrade and Jonas, 2014; Chung et al., 2020). In this study, we employed the use of single-channel cortical EEG recordings to measure generalized seizure activity. Our use of surface EEG did not allow sufficient spatial resolution to identify regional differences in spiking. This technique did, however, allow for analysis of distinct frequency bands in which we demonstrated altered delta/theta frequency band power and peak frequency shift during the ictal phase of convulsive seizures in tau^−/−^ mice that may correlate with modified inhibitory synaptic transmission.

Synaptic reorganization among DGCs occurs in models of acquired TLE, possibly contributing to regional excitability that supports seizures (Buckmaster and Dudek, 1997; Buckmaster et al., 2002; Winokur et al., 2004) and we hypothesized that expression of tau was involved in the synaptic reorganization associated with the development of spontaneous seizures. Even so, we have only a limited understanding of how tau expression alters synaptic transmission in the local dentate gyrus circuit in the absence of additional pathologies (Roberson et al., 2007; Roberson et al., 2011). We found that sEPSC event frequency was not significantly different between mice lacking tau expression and wildtype controls normally, similar to our previous study (Cloyd et al., 2021). After TLE developed, sEPSC frequency was increased similarly in both wildtype and tau^−/−^ mice, suggesting that tau expression does not alter the excitatory synaptic reorganization among DGCs that develops during epileptogenesis in this model. Congruently, we examined hippocampal dispersion associated with TLE development in both genotypes of mice and did not observe any obvious differences between tau^−/−^ mice or wildtype controls. Additionally, hippocampal dispersion was exclusively observed in mice of both genotypes that developed SRS, regardless of whether IHK injection evoked SE, suggesting hippocampal dispersion is associated with TLE development in the IHK model and not kainate toxicity or convulsive SE alone.

Being that IHK induced epileptogenesis may affect additional circuits outside of the dentate gyrus (Sheybani et al., 2018; Fernandez et al., 2022; Franz et al., 2023), further studies are required to elucidate potential differences in excitatory synaptic transmission in other regions that may contribute to modified epileptogenesis after IHK in mice lacking tau expression. For example, global tau ablation in excitatory neurons has been shown to reduce evoked seizure expression after pentylenetetrazole (PTZ) injection in mice (Shao et al., 2022) and deletion of tau reduces spontaneous action potential firing in pyramidal cells in the somatosensory cortex (Chang et al., 2021). Even so, our results do not implicate a role for tau protein in the excitatory synaptic reorganization in the local dentate gyrus circuit that is associated with epileptogenesis.

We found that sIPSC frequency in DGCs ipsilateral to the IHK lesion was reduced similarly in tau^−/−^ and wildtype controls, consistent with either a loss of inhibitory neurons or a reduction in their ability to release GABA in the dentate gyrus (Kobayashi and Buckmaster, 2003). Differently, sIPSC frequency was increased contralateral to the IHK lesion in tau^−/−^ mice that developed TLE compared to vehicle-treated tau^−/−^ mice. It seems possible that this increase in synaptic inhibition could inhibit and/or modify SRS generation, especially seizures that secondarily generalize to involve the hemisphere contralateral to the presumptive seizure initiation zone and may be responsible for reduced epileptiform activity and duration of spontaneous seizures after development of TLE. Axon sprouting and synaptic reorganization of surviving interneurons is associated with development of TLE in rodent models of acquired TLE (Buckmaster and Wen, 2011; Peng et al., 2013; Kang et al., 2022; Lee et al., 2024), and similar or more robust synaptic reorganization may occur in contralateral inhibitory circuits in tau^−/−^ mice. DGCs, including adult born DGCs, form new connections with interneurons after TLE development in multiple models (Buckmaster et al., 2002; Halabisky et al., 2010; Butler et al., 2017; Kang et al., 2022). The restorative increase in synaptic inhibition that occurs contralateral to IHK lesion in the absence of tau expression implies that tau protein may normally suppress this process, but further studies to test this hypothesis directly are needed.

We found that sIPSC event frequency in DGCs was constitutively lower in naïve tau^−/−^ mice. This suggests that tau protein normally plays a role in regulating local inhibitory synaptic transmission in the dentate gyrus. Overexpression of phosphorylated tau suppresses GABAergic neurotransmission and alters intrinsic properties of GABAergic neurons within the dentate gyrus and area CA1 in mice (Zheng et al., 2020). Lack of tau expression in the somatosensory cortex demonstrates a similar dysfunction in inhibitory synaptic transmission (Chang et al., 2021), but whether interneurons in the dentate gyrus are similarly affected by deletion of tau is unclear. Our results identify a novel potential function of tau protein in local inhibitory circuitry in the dentate gyrus, which has not been shown previously.

This study highlights the impact of tau expression on the development of acquired TLE and uncovers potential inhibitory synaptic mechanisms in the dentate gyrus that may underlie tau’s effects on seizures. Here we show that deletion of tau suppresses individual seizures but does not protect against the process of epileptogenesis, highlighting that mice lacking tau expression develop acquired TLE, which has not been demonstrated previously. This study also identifies effects of tau expression on inhibitory synaptic reorganization in the dentate gyrus after epileptogenesis occurs, which may contribute to differential seizure expression in tau^−/−^ mice.

## Significance statement

This study highlights the impact of tau expression on the development of acquired TLE. TLE is the most common focal epilepsy in adults and there are currently few treatment options for patients with drug-resistant TLE, so developing therapeutic targets is necessary to prevent epileptogenesis and/or treat seizures. A lack of tau expression confers resistance to some genetic epilepsies and evoked seizures in rodent models, but effects of tau on acquired epileptogenesis are not well described and the etiologies of evoked seizures and acquired epileptogenesis are vastly different. By examining how lack of tau expression modifies spontaneous seizure development after evoked SE, our results highlight how deletion of tau is not effective in preventing epileptogenesis in a mouse model of TLE, but tau may influence seizure expression via mechanisms involving inhibitory circuitry in the dentate gyrus.

## Data Availability

The raw data supporting the conclusions of this article will be made available by the authors, without undue reservation.
