# Supplementary material for: Differential effects of tau expression on seizures and epileptogenesis in a mouse model of temporal lobe epilepsy
Source: Front Syst Neurosci. 2025 Dec 18;19:1693339. doi: 10.3389/fnsys.2025.1693339 (PMC12756393; doi:10.3389/fnsys.2025.1693339)
Supplement: Supplementary file 1 [file Table_1.DOCX]

**Supplemental Table 1.** Sex differences

| **Seizure Distribution** | | | | | | | | |
| --- | --- | --- | --- | --- | --- | --- | --- | --- |
| **WT (N=49)** | **Male (N=23)** | | | | **Female (N=26)** | | | |
| **Grouped: p=0.39** | No SE:No SRS | | N=3 | | No SE:No SRS | | N=2 | |
| **SE: p=0.77** | No SE:SRS | | N=0 | | No SE:SRS | | N=2 | |
| **SRS: p=0.87** | SE: No SRS | | N=1 | | SE: No SRS | | N=3 | |
|  | SE:SRS | | N=19 | | SE:SRS | | N=19 | |
| **Tau^-/-^ (N=45)** | **Male (N=21)** | | | | **Female (N=24)** | | | |
| **Grouped: p<0.05** | No SE:No SRS | | N=9 | | No SE:No SRS | | N=11 | |
| **SE: p=0.14** | No SE:SRS | | N=6 | | No SE:SRS | | N=2 | |
| **SRS: p=0.31** | SE: No SRS | | N=5 | | SE: No SRS | | N=2 | |
|  | SE:SRS | | N=1 | | SE:SRS | | N=10 | |
| **sEPSC Frequency** | | | | | | | | |
| **WT Veh (N=11)** | | **Male (N=8)** | | | | **Female (N=3)** | | |
| **Mean ± SEM** | | **Frequency** | | **Amplitude** | | **Frequency** | | **Amplitude** |
| **Ipsilateral** | | 0.23 ± 0.03 (n=16) | | 14.72 ± 1.29 | | 0.29 ± 0.04 (n=6) | | 14.55 ± 1.77 |
| **Contralateral** | | 0.22 ± 0.03 (n=16) | | 14.01 ± 0.96 | | 0.25 ± 0.07 (n=6) | | 12.42 ± 1.83 |
| **WT IHK (N=13)** | | **Male (N=8)** | | | | **Female (N=5)** | | |
| **Mean ± SEM** | | **Frequency** | | **Amplitude** | | **Frequency** | | **Amplitude** |
| **Ipsilateral** | | 0.77 ± 0.09 (n=19) | | 14.06 ± 0.83 | | 0.75 ± 0.16 (n=16) | | 12.61 ± 0.81 |
| **Contralateral** | | 0.29 ± 0.07 (n=8) | | 12.17 ± 0.80 | | 0.67 ± 0.18 (n=6) | | 15.07 ± 2.55 |
| **Tau^-/-^ Veh (N=10)** | | **Male (N=8)** | | | | **Female (N=2)** | | |
| **Mean ± SEM** | | **Frequency** | | **Amplitude** | | **Frequency** | | **Amplitude** |
| **Ipsilateral** | | 0.25 ± 0.03 (n=14) | | 12.38 ± 1.19 | | 0.34 ± 0.12 (n=4) | | 8.638 ± 0.52 |
| **Contralateral** | | 0.30 ± 0.02 (n=15) | | 12.23 ± 0.87 | | 0.38 ± 0.03 (n=4) | | 9.045± 0.55 |
| **Tau^-/-^ IHK (N=10)** | | **Male (N=4)** | | | | **Female (N=5)** | | |
| **Mean ± SEM** | | **Frequency** | | **Amplitude** | | **Frequency** | | **Amplitude** |
| **Ipsilateral** | | 0.67 ± 0.12 (n=13) | | 12.56 ± 1.09 | | 0.78 ± 0.16 (n=13) | | 15.10 ± 1.16 |
| **Contralateral** | | 0.41 ± 0.14 (n=7) | | 9.71 ± 1.12 | | 0.54 ± 0.13 (n=11) | | 13.61 ± 1.35 |
| **sIPSC Frequency** | | | | | | | | |
| **WT Veh (N=7)** | | **Male (N=5)** | | | | **Female (N=2)** | | |
| **Mean ± SEM** | | **Frequency** | | **Amplitude** | | **Frequency** | | **Amplitude** |
| **Ipsilateral** | | 0.87 ± 0.11 (n=13) | | 23.327 ± 1.83 | | 1.07 ± 0.15 (n=8) | | 31.05 ± 2.72 |
| **Contralateral** | | 0.65 ± 0.15 (n=10) | | 22.25 ± 0.14 | | 0.88 ± 2.94 (n=4) | | 27.35 ± 6.83 |
| **WT IHK (N=5)** | | **Male (N=3)** | | | | **Female (N=2)** | | |
| **Mean ± SEM** | | **Frequency** | | **Amplitude** | | **Frequency** | | **Amplitude** |
| **Ipsilateral** | | 0.08 ± 0.02 (n=11) | | 36.85 ± 5.49 | | 0.06 ± 0.03 (n=5) | | 37.59 ± 3.24 |
| **Contralateral** | | 0.61 ± 0.08 (n=10) | | 25.52 ± 0.15 | | 0.62 ± 0.13 (n=5) | | 36.91 ± 6.12 |
| **Tau^-/-^ Veh (N=8)** | | **Male (N=5)** | | | | **Female (N=3)** | | |
| **Mean ± SEM** | | **Frequency** | | **Amplitude** | | **Frequency** | | **Amplitude** |
| **Ipsilateral** | | 0.40 ± 0.06 (n=12) | | 25.23 ± 3.52 | | 0.73 ± 0.01 (n=2) | | 32.85 ± 3.35 |
| **Contralateral** | | 0.38 ± 0.05 (n=10) | | 27.62 ± 2.67 | | 0.41 ± 0.10 (n=5) | | 35.30 ± 4.90 |
| **Tau^-/-^ IHK (N=6)** | | **Male (N=3)** | | | | **Female (N=3)** | | |
| **Mean ± SEM** | | **Frequency** | | **Amplitude** | | **Frequency** | | **Amplitude** |
| **Ipsilateral** | | 0.22 ± 0.05 (n=11) | | 37.12 ± 2.76 | | 0.05 ± 0.01 (n=6) | | 45.39 ± 6.28 |
| **Contralateral** | | 0.90 ± 0.16 (n=11) | | 28.11 ± 2.79 | | 0.80 ± 0.16 (n=8) | | 38.81 ± 3.75 |
