# Supplementary material for: Differential effects of tau expression on seizures and epileptogenesis in a mouse model of temporal lobe epilepsy
Source: Front Syst Neurosci. 2025 Dec 18;19:1693339. doi: 10.3389/fnsys.2025.1693339 (PMC12756393; doi:10.3389/fnsys.2025.1693339)
Supplement: Supplementary file 2 [file Table_2.DOCX]

**Supplemental Table 2.** Statistical analysis for all figures

| **Figure** | **Structure** | **Type of test** | **Results** |
| --- | --- | --- | --- |
| Fig. 1A | Nonparametric | Chi-square | X^2^ = 9.085  p = 0.0026 |
| Fig. 1B | Normal | Unpaired t test (two tailed) | t = 2.490, df = 16  p = 0.0241 |
| Fig. 1C | Normal | Unpaired t test (two tailed) | t = 0.362, df = 7  p = 0.497 |
| Fig. 1F | Normal | Unpaired t test (two tailed) | t = 1.705, df = 74  p = 0.0923 |
| Fig. 1G | Normal | Unpaired t test (two tailed) | t = 1.221, df = 104  p = 0.2250 |
| Fig. 1H | Normal | Unpaired t test (two tailed)  Two-way ANOVA | Periodogram (AUC)  t = 1.818, df =52  p = 0.0749  Power Bands  Sidak’s multiple comparisons test  WT vs. Tau^-/-^  Delta, p<0.0001  Theta, p=0.0094  Alpha, p=-0.7398  Sigma, p=0.6885  Beta, p=0.9686  Gamma, p=0.9717   \| ANOVA table \| SS \| DF \| MS \| F \| \| P-value \| \| --- \| --- \| --- \| --- \| --- \| --- \| --- \| \| Row Factor \| 1.96e-17 \| 1 \| 1.96e-17 \| F (1, 294) = 45.86 \| \| p<0.0001 \| \| Column Factor \| 1.58e-17 \| 5 \| 1.58e-17 \| F (5, 294) = 36.86 \| \| p < 0.0001 \| \| Residual \| 1.23e-16 \| 294 \| 4.28e-19 \|  \| \|  \| \|  \|  \|  \| \| \| |
| Fig. 2A | Nonparametric | Chi-square | t = 0.2250, df = 104  p = 0.2250 |
| Fig. 2B | Nonparametric | Chi-square | X^2^ = 0.003  p = 0.9583 |
| Fig. 2C | Normal | Two-way ANOVA | \| ANOVA table \| SS \| DF \| MS \| F \| P-value \| \| --- \| --- \| --- \| --- \| --- \| --- \| \| Interaction \| 9.675 \| 1 \| 9.675 \| F (1, 54) = 1.298 \| p=0.2596 \| \| Row Factor \| 5.615 \| 1 \| 5.615 \| F (1, 54) = 0.7533 \| p=0.3893 \| \| Column Factor \| 0.02839 \| 1 \| 0.02839 \| F (1, 54) = 0.003809 \| p=0.9510 \| \| Residual \| 402.5 \| 54 \| 7.454 \|  \|  \|   Tukey’s multiple-comparisons test  WT:No SE : SRS vs. WT:SE : SRS, p = 0.8897  WT:No SE : SRS vs. Tau-/-:No SE : SRS, p = 0.6909  WT:No SE : SRS vs. Tau-/-:SE : SRS, p = 0.9539  WT:SE : SRS vs. Tau-/-:No SE : SRS, p = 0.8058  WT:SE : SRS vs. Tau-/-:SE : SRS, p = 0.9874  Tau-/-:No SE : SRS vs. Tau-/-:SE : SRS, p = 0.7588 |
| Fig. 4C | Normal | Unpaired t test (two tailed) | t = 4.828, df = 17  p = 0.0002 |
| Fig. 4D | Normal | Unpaired t test (two tailed) | t = 2.340, df = 17  p = 0.0317 |
| Fig. 4E | Normal | Unpaired t test (two tailed) | Periodogram (AUC)  t = 2.382, df =19  p = 0.028  Power Bands  Sidak’s multiple comparisons test  WT vs. Tau^-/-^  Delta, p>0.9999  Theta, p>0.9999  Alpha, p=-0.1712  Sigma, p>0.9999  Beta, p>0.9999  Gamma, p>0.9999   \| ANOVA table \| SS \| DF \| MS \| F \| P-value \| \| --- \| --- \| --- \| --- \| --- \| --- \| \| Row Factor \| 2.21e-19 \| 1 \| 2.21e-19 \| F (1, 114) = 0.8105 \| p=0.3699 \| \| Column Factor \| 2.47e-18 \| 5 \| 4.94e-19 \| F (5, 114) = 1.810 \| P=0.1163 \| \| Residual \| 3.11e-17 \| 114 \| 2.73e-19 \|  \|  \| |
| Fig. 5D | Normal | Two-way ANOVA | \| ANOVA table \| SS \| DF \| MS \| F \| P-value \| \| --- \| --- \| --- \| --- \| --- \| --- \| \| Row Factor \| 4.443 \| 45 \| 0.09872 \| F (45, 123) = 1.298 \| p=0.9074 \| \| Column Factor \| 7.709 \| 5 \| 1.542 \| F (45, 123) = 11.04 \| p < 0.0001 \| \| Residual \| 17.17 \| 123 \| 0.1396 \|  \|  \|   Tukey’s multiple-comparisons test   \| Vehicle WT vs. Vehicle Tau^-/-^, p = 0.9777 \| \| --- \| \| Vehicle WT vs. Ipsilateral WT, p < 0.0001 \| \| Vehicle WT vs. Ipsilateral Tau^-/-^, p < 0.0001 \| \| Vehicle WT vs. Contralateral WT, p = 0.2891 \| \| Vehicle WT vs. Contralateral Tau^-/-^, p = 0.4066 \| \| Vehicle Tau^-/-^ vs. Ipsilateral WT, p < 0.0001 \| \| Vehicle Tau^-/-^ vs. Ipsilateral Tau^-/-^, p = 0.0004 \| \| Vehicle Tau^-/-^ vs. Contralateral WT, p = 0.5946 \| \| Vehicle Tau^-/-^ vs. Contralateral Tau^-/-^, p = 0.7583 \| \| Ipsilateral WT vs. Ipsilateral Tau^-/-^, p = 0.9952 \| \| Ipsilateral WT vs. Contralateral WT, p = 0.2259 \| \| Ipsilateral WT vs. Contralateral Tau^-/-^, p = 0.0512 \| \| Ipsilateral Tau^-/-^ vs. Contralateral WT, p = 0.4843 \| \| Ipsilateral Tau^-/-^ vs. Contralateral Tau^-/-^, p = 0.1830 \| \| Contralateral WT vs. Contralateral Tau^-/-^, p = 0.993 \| |
| Fig. 5E | Normal | Two-way ANOVA | \| ANOVA table \| SS \| DF \| MS \| F \| P-value \| \| --- \| --- \| --- \| --- \| --- \| --- \| \| Row Factor \| 882.6 \| 45 \| 19.61 \| F (45, 123) = 1.645 \| p = 0.0173 \| \| Column Factor \| 203.5 \| 5 \| 40.70 \| F (45, 123) = 3.414 \| p = 0.0064 \| \| Residual \| 1431 \| 123 \| 0.1396 \|  \|  \|   Tukey’s multiple-comparisons test   \| Vehicle WT vs. Vehicle Tau^-/-^, p = 0.0534 \| \| --- \| \| Vehicle WT vs. Ipsilateral WT, p = 0.9994 \| \| Vehicle WT vs. Ipsilateral Tau^-/-^, p = 0.9802 \| \| Vehicle WT vs. Contralateral WT, p = 0.0989 \| \| Vehicle WT vs. Contralateral Tau^-/-^, p = 0.1264 \| \| Vehicle Tau^-/-^ vs. Ipsilateral WT, p = 0.1549 \| \| Vehicle Tau^-/-^ vs. Ipsilateral Tau^-/-^, p = 0.4352 \| \| Vehicle Tau^-/-^ vs. Contralateral WT, p = 0.9910 \| \| Vehicle Tau^-/-^ vs. Contralateral Tau^-/-^, p = 0.9997 \| \| Ipsilateral WT vs. Ipsilateral Tau^-/-^, p = 0.9987 \| \| Ipsilateral WT vs. Contralateral WT, p = 0.1729 \| \| Ipsilateral WT vs. Contralateral Tau^-/-^, p = 0.2261 \| \| Ipsilateral Tau^-/-^ vs. Contralateral WT, p = 0.3389 \| \| Ipsilateral Tau^-/-^ vs. Contralateral Tau^-/-^, p = 0.4391 \| \| Contralateral WT vs. Contralateral Tau^-/-^, p = 0.9996 \| |
| Fig. 6D | Normal | Two-way ANOVA | \| ANOVA table \| SS \| DF \| MS \| F \| P-value \| \| --- \| --- \| --- \| --- \| --- \| --- \| \| Row Factor \| 4.211 \| 34 \| 0.1239 \| F (34, 91) = 1.385 \| p = 0.0878 \| \| Column Factor \| 11.50 \| 5 \| 2.299 \| F (5, 91) = 29.10 \| p < 0.0001 \| \| Residual \| 7.825 \| 91 \| 0.086 \|  \|  \|   Tukey’s multiple-comparisons test   \| Vehicle WT vs. Vehicle Tau^-/-^, p < 0.0001 \| \| --- \| \| Vehicle WT vs. Ipsilateral WT, p < 0.0001 \| \| Vehicle WT vs. Ipsilateral Tau^-/-^, p < 0.0001 \| \| Vehicle WT vs. Contralateral WT, p = 0.0391 \| \| Vehicle WT vs. Contralateral Tau^-/-^, p = 0.9997 \| \| Vehicle Tau^-/-^ vs. Ipsilateral WT, p = 0.0.253 \| \| Vehicle Tau^-/-^ vs. Ipsilateral Tau^-/-^, p = 0.0151 \| \| Vehicle Tau^-/-^ vs. Contralateral WT, p = 0.4987 \| \| Vehicle Tau^-/-^ vs. Contralateral Tau^-/-^, p < 0.0001 \| \| Ipsilateral WT vs. Ipsilateral Tau^-/-^, p > 0.9999 \| \| Ipsilateral WT vs. Contralateral WT, p = 0.0003 \| \| Ipsilateral WT vs. Contralateral Tau^-/-^, p < 0.0001 \| \| Ipsilateral Tau^-/-^ vs. Contralateral WT, p = 0.0002 \| \| Ipsilateral Tau^-/-^ vs. Contralateral Tau^-/-^, p < 0.0001 \| \| Contralateral WT vs. Contralateral Tau^-/-^, p = 0.1036 \| |
| Fig. 6E | Normal | Two-way ANOVA | \| ANOVA table \| SS \| DF \| MS \| F \| P-value \| \| --- \| --- \| --- \| --- \| --- \| --- \| \| Row Factor \| 4160 \| 34 \| 122.4 \| F (34, 91) = 1.225 \| p = 0.2215 \| \| Column Factor \| 1733 \| 5 \| 346.5 \| F (5, 91) = 3.470 \| p = 0.0064 \| \| Residual \| 9187 \| 91 \| 99.85 \|  \|  \|   Tukey’s multiple-comparisons test   \| Vehicle WT vs. Vehicle Tau^-/-^, p = 0.6096 \| \| --- \| \| Vehicle WT vs. Ipsilateral WT, p = 0.0517 \| \| Vehicle WT vs. Ipsilateral Tau^-/-^, p = 0.0057 \| \| Vehicle WT vs. Contralateral WT, p = 0.7179 \| \| Vehicle WT vs. Contralateral Tau^-/-^, p = 0.0789 \| \| Vehicle Tau^-/-^ vs. Ipsilateral WT, p = 0.5832 \| \| Vehicle Tau^-/-^ vs. Ipsilateral Tau^-/-^, p = 0.1861 \| \| Vehicle Tau^-/-^ vs. Contralateral WT, p < 0.9999 \| \| Vehicle Tau^-/-^ vs. Contralateral Tau^-/-^, p = 0.7527 \| \| Ipsilateral WT vs. Ipsilateral Tau^-/-^, p = 0.9899 \| \| Ipsilateral WT vs. Contralateral WT, p = 0.7847 \| \| Ipsilateral WT vs. Contralateral Tau^-/-^, p = 0.9992 \| \| Ipsilateral Tau^-/-^ vs. Contralateral WT, p = 0.3987 \| \| Ipsilateral Tau^-/-^ vs. Contralateral Tau^-/-^, p = 0.9118 \| \| Contralateral WT vs. Contralateral Tau^-/-^, p = 0.9114 \| |
